# Ecological features and sozological assessment of *Unio crassus* in watercourses of the right-bank Ukraine in the context of EU environmental directives implementation

**DOI:** 10.1186/s12862-026-02568-y

**Published:** 2026-08-01

**Authors:** Larysa M. Shevchuk, Liliia V. Bylyna, Andrii V. Shevchuk, Tetiana A. Vakaliuk, Serhiy O. Semerikov

**Affiliations:** 1 Zhytomyr Polytechnic State University, 103 Chudnivsyka Str., Zhytomyr, 10005 Ukraine; 2 Berdychiv Medical College, 14 Shevchenka Str., Berdychiv, 13300 Ukraine; 3 Institute for Digitalisation of Education of the NAES of Ukraine, 9 M. Berlynskoho Str., Kyiv, 04060 Ukraine; 4 Kryvyi Rih State Pedagogical University, 54 Universytetskyi Ave., Kryvyi Rih, 50086 Ukraine; 5 Academy of Cognitive and Natural Sciences, 54 Universytetskyi Ave., Kryvyi Rih, 50086 Ukraine; 6 Center for Information-Analytical and Technical Support of Nuclear Power Facilities Monitoring of the NAS of Ukraine, 34a Palladin Ave., Kyiv, 03142 Ukraine

**Keywords:** *Unio crassus*, Freshwater bivalves, Population structure, Conservation status, Bioindication, Ukraine

## Abstract

The current status of *Unio crassus* populations in the watercourses of the right-bank Ukraine was studied. Of 191 localities surveyed, the species was found at 30, across four river basins: the Danube, Dniester and Southern Bug basins and the Prypiat sub-basin. Co-occurrence of *U. crassus* with other Unionidae was analysed using the Jaccard and Sørensen–Dice indices; the highest values were found with *U. pictorum* ($K_J$ = 0.59) and *U. tumidus* ($K_J$ = 0.52), indicating similar habitat requirements. Comparative assessment of the combined demographic and morphometric indicators distinguished three population groups. The populations of the Ubort (Sushchany) and Stalineshti (Mamalyha) rivers showed the most favourable demographic and morphometric indicators and are regarded as candidate donor populations for species recovery. Morphometric analysis revealed variability in shell size, with the largest shells in the Stalineshti River population (mean shell length *68.86 ± 2.52* mm). A comparison of the Prypiat sub-basin between 2007 and 2011 and 2020–2024 indicated an overall decline in occurrence (from 40.6% to 20.0% of sites; Fisher’s exact test, *p=0.071*), including loss of detections in the Noryn River and a reduction in the Ubort River; small, uneven samples and differences in survey effort mean these trends should be interpreted with caution. The results can inform the planning of protected areas, particularly Emerald Network sites, the development of species management plans, and the adaptation of European *U. crassus* conservation experience to Ukraine.

## Introduction

Threats to populations of bivalve molluscs (Mollusca, Bivalvia) have been observed worldwide for a long time. Molluscs account for 42% of species that have gone extinct since 1500, including 31 species of bivalves. As of 2015, 44% of freshwater bivalve species were classified as endangered or near-endangered [1, 2]. In Europe, three species of bivalves are critically endangered (CR: river pearl mussel *Margaritifera margaritifera, Pseudunio auricularius* and *Unio gibbus*), two species are endangered (EN: *Potomida littoralis* and *U. crassus*), and four species are vulnerable (VU: *Anodonta cygnea, Pseudanodonta complanata, U. delphinus* and *U. mancus*) (according to the IUCN Red List) [3].

*U. crassus* is distributed across Europe and Western and Northern Asia and was a common inhabitant of rivers and streams until the end of the nineteenth century. In the second half of the twentieth century, however, its range in Central Europe contracted by about 50%. The main drivers of this decline were industrial development, river canalisation and increasing water pollution [4, 5], together with changes in the composition of the fish community that reduced the availability of the host fish on which the glochidial larvae depend for metamorphosis [6]. In addition, the introduction of muskrat (*Ondatra zibethicus*) in Europe has had a negative impact on local populations of *U. crassus*, as this rodent, despite its predominantly plant-based diet, actively consumes bivalves [7]. It is believed that viable populations have survived in Eastern European river systems. However, complete information is not yet available, as the species is not subject to regular monitoring observations here.

Significant declines in thick-shelled river mussel (*Unio crassus*) populations were observed in many European countries in the 1980s. In 2014, the species was included in the IUCN Red List as Endangered (EN) and the European Red List (Vulnerable), but the degree of its threat varies in different parts of Europe and depends on the country [8, 9]. The species is also included in Resolution 6 of the Bern Convention and the Red Data Book of Ukraine (2021 edition).

The thick-shelled river mussel (*U. crassus*) is included in Annexes II and IV of the 1992 European Council Habitats Directive (92/43/EEC), which obliges EU countries to develop conservation plans, designate protected areas for representative populations, and monitor the species’ conservation status.

In the European Union, a comprehensive system of *U. crassus* conservation is being implemented within the framework of LIFE projects funded by the European Commission [10]. The key areas of these projects are population assessment, habitat improvement, and reintroduction of the species.

The largest is the German LIFE BACHMUSCHEL project (2023–2032), which includes annual monitoring of populations in the Elbe, Havel and Spree rivers, focusing on restoring natural channels and regulating the hydrological regime. In Luxembourg (LIFE *Unio crassus* project), a program of artificial breeding and reintroduction of molluscs in the Sauer and Our rivers is being implemented with further monitoring of their survival.

Significant progress has been made in Sweden, where the UC4LIFE project (2012–2016) restored the species’ habitats along a 267 km stretch of rivers. Comprehensive studies in Serbia (1953–2019) have identified key environmental factors affecting *U. crassus* populations: altitude gradient, substrate characteristics, and nitrate levels in water.

Thus, the European experience demonstrates the effectiveness of an integrated approach to *U. crassus* conservation that combines monitoring, habitat restoration, and reintroduction of the species [6]. Monitoring programs for the species range from annual surveys to long-term projects with an interval of 3–5 years, depending on conservation priorities and national characteristics.

The monitoring methodology covers a wide range of parameters. The fundamental indicators are population size and density, which allow us to assess the viability of a species in a particular environment. The demographic structure, particularly the age distribution, indicates successful reproduction and population stability. Physiological parameters of the molluscs provide information on the quality of the water environment and anthropogenic load. Particular attention is paid to a comprehensive analysis of the species’ habitat and the state of biodiversity of related species. Modern monitoring programs also include genetic research to assess population structure and identify potential threats to genetic diversity. The experience of European countries demonstrates different approaches to the organisation of monitoring. In Germany and Poland [11, 12], mussel research has contributed to implementing effective water management measures and restoring degraded river ecosystems.

Generally, *U. crassus* is a long-lived mollusc (with a maximum lifespan of 15–30 years in European waters) and a typical rheophile that prefers clean, well-oxygenated water with low levels of organic pollution. Its populations are significantly affected by pollution caused by nitrates, pesticides, heavy metals, siltation of the bottom, and disturbance of the natural channel regime. These features make the species a reliable bioindicator of water quality.

Research and conservation of *U. crassus* in Ukraine are particularly relevant to European integration processes and the implementation of European environmental legislation [13].

The lack of systematic monitoring of *U. crassus* in Ukraine creates significant gaps in understanding the current status of the species and makes it impossible to plan effective conservation measures. This becomes especially critical in the context of the Emerald Network [14], where the availability of scientifically based data on the status of populations and their spatial distribution is a prerequisite for identifying areas in need of protection.

The species demonstrates high sensitivity to anthropogenic environmental changes, acting as a natural biofilter and playing an important role in maintaining the ecological balance of aquatic ecosystems. Its presence and viability of populations can serve as reliable indicators of the general condition of river biocenoses.

Implementing the European experience of *U. crassus* monitoring into Ukrainian conservation practice will not only standardise methodological approaches but also integrate national data into the pan-European observation system. This will create the basis for the development of effective management plans [15] and the implementation of scientifically based conservation measures.

The results of systematic research will serve as the basis for identifying priority conservation areas, planning the restoration of degraded river ecosystems, and developing comprehensive measures to preserve the biodiversity of Ukraine’s aquatic ecosystems. This approach will ensure the fulfilment of international obligations to protect the species and facilitate the integration of the national nature conservation system into the European environmental space.

## Material and methods

### Study area and sampling design

Monitoring surveys were conducted in the watercourses of the right-bank Ukraine, covering four river basins – the Danube, Dniester and Southern Bug basins and the Prypiat sub-basin of the Dnipro basin – as well as the Western Bug basin. A total of 191 sites were surveyed. The principal survey was carried out from 2007 to 2018; in 2020–2024, a repeat survey of the Prypiat sub-basin was conducted to assess the dynamics of the populations. At each site, molluscs were collected by hand during the warm season (May to September). The geographic coordinates of every sampling site were recorded (Table 1), and occurrence records were deposited in MolluscaBase [16]. All fieldwork complied with the bioethical standards for research on wildlife and with the national environmental legislation of Ukraine, as *U. crassus* is listed in the Red Data Book of Ukraine.

**Table 1:** Occurrence sites of *U. crassus* in the watercourses of the right-bank Ukraine, with geographic coordinates

River	Occurrence site	Coordinates
**Danube basin**		
Borzhava	Vilkhivka	$48^{\circ}$15’49” N, $23^{\circ}$4’32” E
Borzhava	Vary	$48^{\circ}$7’17” N, $22^{\circ}$42’39” E
Irshava	Siltse	$48^{\circ}$17’14” N, $22^{\circ}$59’50” E
Latorytsia	Mukachevo	$48^{\circ}$26’14” N, $22^{\circ}$41’18” E
Latorytsia	Solomonove	$48^{\circ}$27’13” N, $22^{\circ}$9’57” E
Latorytsia	Stare Davydkovo	$48^{\circ}$27’52” N, $22^{\circ}$38’2” E
Latorytsia	Chabanivka	$48^{\circ}$29’18” N, $22^{\circ}$32’51” E
Apshytsia	Hrushovo	$48^{\circ}$0’24” N, $23^{\circ}$45’44” E
Siret	Staryi Vovchynets	$48^{\circ}$0’50” N, $25^{\circ}$57’51” E
Stalineshti	Mamalyha	$48^{\circ}$15’20” N, $26^{\circ}$35’14” E
Uzh (Danube)	Uzhhorod	$48^{\circ}$37’7” N, $22^{\circ}$17’55” E
**Dniester basin**		
Dniester	Zalishchyky	$48^{\circ}$38’53” N, $25^{\circ}$44’15” E
Hnizna	Terebovlia	$49^{\circ}$18’25” N, $25^{\circ}$42’4” E
Murafa	Bila	$48^{\circ}$15’6” N, $28^{\circ}$13’15” E
**Southern Bug basin**		
Southern Bug	Lupolove	$48^{\circ}$8’34” N, $30^{\circ}$18’11” E
Southern Bug	Pivdennoukrainsk	$47^{\circ}$49’0” N, $31^{\circ}$10’59” E
Inhul	Sofiivka	$47^{\circ}$41’6” N, $32^{\circ}$22’36” E
**Prypiat sub-basin**		
Sluch	Baranivka	$50^{\circ}$17’38” N, $27^{\circ}$40’7” E
Sluch	Chyzhivka	$50^{\circ}$40’6” N, $27^{\circ}$37’28” E
Horyn	Horodets	$51^{\circ}$17’6” N, $26^{\circ}$21’55” E
Ubort	Olevsk	$51^{\circ}$13’26” N, $27^{\circ}$38’54” E
Ubort	Sushchany	$51^{\circ}$17’56” N, $27^{\circ}$45’1” E
Ubort	Khochyno	$51^{\circ}$27’38” N, $27^{\circ}$52’53” E
Styr	Maiunychi	$51^{\circ}$14’59” N, $25^{\circ}$56’51” E
Noryn	Bohdanivka	$51^{\circ}$17’18” N, $28^{\circ}$58’29” E
Zherev	Ihnatpil	$51^{\circ}$8’37” N, $28^{\circ}$44’2” E
Smilka	Smolka	$50^{\circ}$34’17” N, $27^{\circ}$36’9” E
Tnia	Molodizhne	$50^{\circ}$27’2”,N, $28^{\circ}$8’20” E
Tnia	Sokoliv	$50^{\circ}$29’43” N, $28^{\circ}$4’6” E
Khomora	Pershotravneve	$50^{\circ}$12’3” N, $27^{\circ}$38’6” E

### Density estimation

Population density was expressed as the number of individuals per square metre (individuals/m$^2$). Molluscs were collected by hand, examining the bottom sediment to approximately a palm’s depth. At each site where the species was present, three replicate 1 m$^2$ quadrats were counted within the occupied patch, and the local density was calculated as the mean of the three replicates. Per-basin density is reported as the mean *±* standard error with the observed range.

### Age and morphometric analysis

The age of each individual was determined by counting the annual growth-cessation (winter) rings on the shell, read on both valves simultaneously to ensure accuracy. Shell length, height and convexity were measured with a vernier calliper to the nearest 0.1 mm. Because of the limited amount of material that could be collected from this protected species, full age and morphometric analyses were possible only for a subset of populations; reported morphometric values are means *±* standard error.

### Community (co-occurrence) analysis

Co-occurrence of *U. crassus* with the other native Unionidae was quantified from the species presence/absence matrix across the surveyed localities using the Jaccard index ($K_J = a/(a+b+c)$) and the Sørensen–Dice index ($K_{SC} = 2a/(2a+b+c)$), where *a* is the number of localities at which both species occur and *b* and *c* are the numbers of localities at which only one of the pair occurs. Both indices range from 0 (no shared localities) to 1 (fully concordant occurrence); higher values indicate more similar habitat occupancy.

### Statistical analysis

Because most population samples were small and unevenly sized, non-parametric methods were used and the results were interpreted conservatively. Differences in age structure among the right-bank river basins were tested with the Kruskal–Wallis test. The change in the occurrence of *U. crassus* in the Prypiat sub-basin between the 2007–2011 and 2020–2024 surveys was tested with Fisher’s exact test on the numbers of occupied versus unoccupied sites. The significance threshold was set at *α = 0.05*. Populations were grouped into three categories according to their demographic and morphometric parameters (population density, age structure, maximum recorded age and shell dimensions) by comparative assessment of the combined indicators. Descriptive statistics were computed in Microsoft Excel; the Kruskal–Wallis test and Fisher’s exact test were performed using standard statistical formulae.

### Sozological assessment

The sozological (conservation) value of each population was assessed from a combination of demographic and morphometric indicators – population density, the balance of the age structure (the number of age classes represented and the presence of younger individuals), maximum longevity and shell size – interpreted against the criteria of the EU Habitats Directive (92/43/EEC) and the Water Framework Directive. Populations that combined comparatively high density, a relatively balanced age structure and large body size were regarded as having the highest sozological value and as candidate donor populations for potential species recovery.

## Results

### Distribution and occurrence

**Fig. 1:**
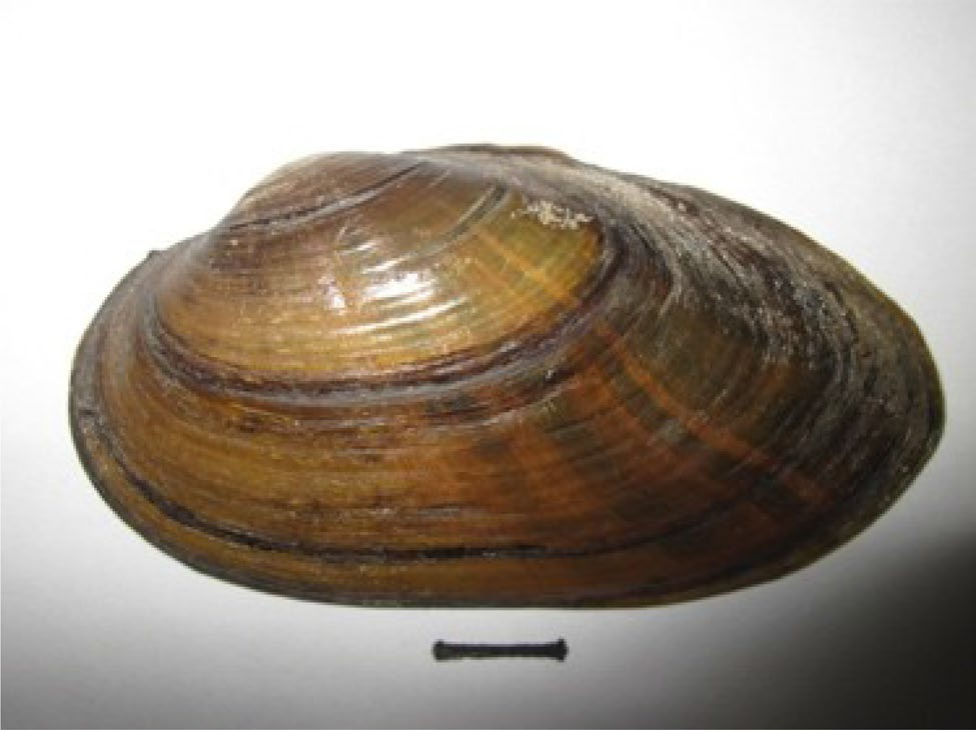
Shells of *U. crassus* (the Stalineshti River, Mamalyha village)

In the water bodies of the right-bank Ukraine, *U. crassus* s. str. was recorded (Fig. 1). Of the 191 sites surveyed, the species was present at 30 sites, distributed across four river basins with characteristic hydrological and hydrochemical features – the Danube, Dniester and Southern Bug basins and the Prypiat sub-basin – and was absent from the Western Bug basin. The present distribution of the species is markedly mosaic.

The four basins differ in their hydrological and hydrochemical character, which is reflected in the within-basin distribution of the species.

The Danube basin covers the mountainous and foothill areas of the Carpathians with fast flows, rocky bottoms, and high oxygen saturation. Slow flow areas are characteristic of the plain section of the Latorytsia. Significant seasonal fluctuations in water level and flood conditions are typical. Here, the species is found mainly in tributaries – the Borzhava, Latorytsia, and Uzh rivers.

The Dniester basin includes mountain streams of the Carpathians and flat areas with moderate flow and sand and gravel substrate. *U. crassus* was recorded in the middle reaches of the Dniester and its tributaries, the Hnizna and Murafa.

The Southern Bug basin is characterised by a flat type with alternating rapids and calm areas. The bottom is mostly rocky and sandy. The species is found in the main channel and the Ingul tributary.

Of the Dnipro basin water bodies, the species was found only in the Prypiat sub-basin. The Prypiat sub-basin is characterised by slow to medium flows that increase on rifts, sandy and silty-sandy bottom sediments, and a high content of humic substances. Here, *U. crassus* was found in the largest number of localities, mainly in the tributaries – the Sluch, Ubort, Horyn and Styr rivers.

**Fig. 2:**
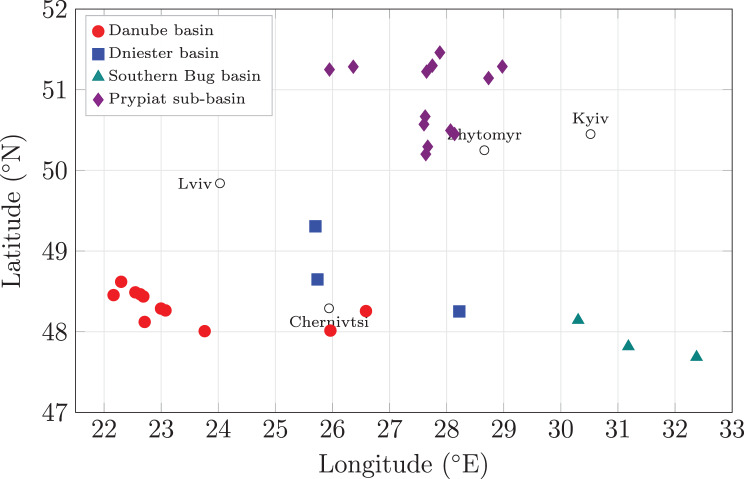
Occurrence sites of *U. crassus* in the right-bank Ukraine, coded by river basin (markers distinguished by both colour and shape). Open circles mark reference cities

In total, *U. crassus* was recorded at 30 occurrence sites across the right-bank Ukraine (Fig. 2; Table 1).

### Frequency of occurrence

**Fig. 3:**
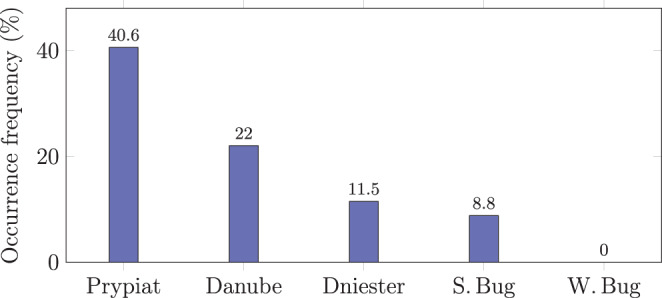
Occurrence frequency (%) of *U. crassus* across river basins

The frequency of occurrence of *U. crassus* across the surveyed right-bank basins (the Danube, Dniester, Southern Bug and Western Bug basins and the Prypiat sub-basin) was highest in the Prypiat sub-basin. As we reported previously [17], the occurrence frequency was 41% in the Prypiat sub-basin (13 of 32 localities surveyed). For comparison, the species was found at 22% of localities in the Danube basin, and at lower frequencies elsewhere: Dniester − 11.54%, Southern Bug − 8.82% (Fig. 3).

### Co-occurrence with other unionidae

The Unionidae assemblages at the *U. crassus* occurrence sites were analysed. Only in the Danube basin was the species found at localities where it was the sole representative of the family (six such localities); at all other sites it co-occurred with other native freshwater mussels (the water bodies of Ukraine host six native unionid species, including *U. crassus*).

According to the Jaccard ($K_J$) and Sørensen–Dice ($K_{SC}$) coefficients, *U. crassus* showed the highest co-occurrence with congeners of its own genus – *U. pictorum* ($K_J$ = 0.59; $K_{SC}$ = 0.74) and *U. tumidus* ($K_J$ = 0.52; $K_{SC}$ = 0.69) (Table 2).

**Table 2:** Coexistence coefficients of *U. crassus* with other species

Species	Shared localities	**Jaccard index (**$K_J$**)**	**Sørensen-Dice index (**$K_{SC}$**)**
*U. tumidus*	24	0.52	0.69
*U. pictorum*	26	0.59	0.74
*A. anatina*	19	0.37	0.54
*A. cygnea*	1	0.01	0.03
*P. complanata*	13	0.23	0.37
*S. woodiana*	1	0.01	0.03

Lower values were found for co-occurrence with *A. anatina* ($K_J$ = 0.37; $K_{SC}$ = 0.54) and *P. complanata* ($K_J$ = 0.23; $K_{SC}$ = 0.37); the low value for the latter is consistent with its generally low frequency of occurrence (about 10%) in Ukrainian water bodies. The minimum values were recorded for *A. cygnea* and *S. woodiana* ($K_J$ = 0.01; $K_{SC}$ = 0.03); *S. woodiana* is an invasive species recorded in Ukraine only in the Danube basin (Table 2).

### Species richness of the assemblages

**Fig. 4:**
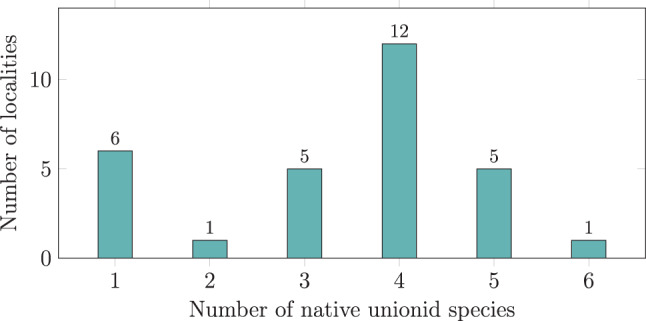
Number of native unionid species recorded at the 30 *U. crassus* occurrence sites

Across the 30 occurrence sites, native unionid species richness ranged from one to six species (Fig. 4). At six sites, all in the Danube basin, *U. crassus* was the sole unionid present. Where it co-occurred with other unionids, the most frequent assemblage comprised four species (12 sites) – typically *U. tumidus*, *U. pictorum* and *A. anatina* together with *U. crassus*; five species were recorded at five sites (with *P. complanata* added to this complex), three species at five sites, and two species at a single site.

The highest richness (six native species) was recorded at the Tnia River (Sokoliv), where *A. cygnea* was also present. Three or more native unionid species occurred together at 23 of the 30 sites (77%).

### Population density

**Fig. 5:**
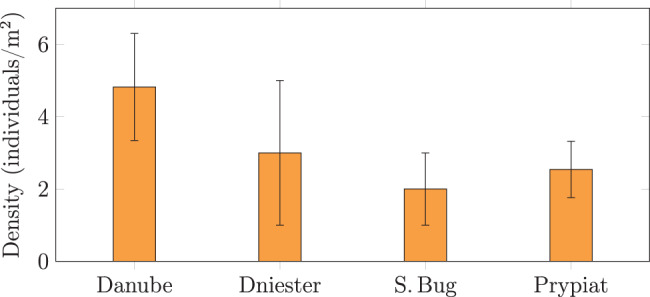
Population density of *U. crassus* by river basin (mean *±* standard error; individuals/m$^2$)

Population density varied among the river basins (Fig. 5); the full analysis across all river basins of Ukraine was reported earlier [17]. The highest mean density was recorded in the Danube basin (*4.82 ± 1.48* individuals/m$^2$; *n=11*), with a maximum of 15 individuals/m$^2$ at a single site. In the Prypiat sub-basin, despite the highest frequency of occurrence, the mean density was *2.54 ± 0.78* individuals/m$^2$ (*n=13*; range 1–10). The remaining basins showed comparable or lower values: Dniester *3.00 ± 2.00* individuals/m$^2$ (*n=3*) and Southern Bug *2.00 ± 1.00* individuals/m$^2$ (*n=3*). The small and uneven numbers of occupied sites per basin preclude a formal statistical comparison of density among basins.

### Age structure

The age structure was examined in detail for the populations from which sufficient material was available (*N=45* individuals in total). Median age did not differ significantly among the right-bank basins (Kruskal–Wallis test, *H=4.47*, $\mathit{df}=3$, *p=0.215*), although this comparison is underpowered given the small and uneven samples (see section “Limitations”).

In the Borzhava River (Vilkhivka), only two individuals (aged 3 and 5 years) were found. Six individuals were collected in the Stalineshti River (Mamalyha), with a predominance of middle-aged individuals (6–7 years) and one 8-year-old individual. Two 5-year-old individuals were collected in the Murafa River (Bila), and a single 8-year-old individual in the Southern Bug River (Lupolove). The Sluch River population (Baranivka) comprised six individuals, the majority (four specimens) belonging to the 4-year age class.

The largest sample (19 individuals) was recorded in the Ubort River (Sushchany), spanning ages 4–7 years with 5–6-year-olds predominating (seven specimens in each class). Three individuals were found in the Styr River (Maiunychi): two 6-year-olds and one 8-year-old. In the Tnia River (Sokoliv), of six individuals most belonged to the younger age classes (3–5 years), with a single 7-year-old.

No individuals younger than two years were recorded at any locality (0 of 45 individuals). This may reflect limited recruitment; however, it should be interpreted cautiously, given the small, non-systematic samples and the absence of sampling specifically targeting juveniles and glochidia (see Limitations).

Middle-aged individuals (5–6 years) dominated, accounting for 57.8% of the individuals examined. The maximum age recorded (8 years) was observed at only three localities: the Stalineshti River (Mamalyha), the Southern Bug River (Lupolove) and the Styr River (Maiunychi). The most balanced age structure – multiple age classes represented together with the largest sample – was found in the Ubort River (Sushchany), which is therefore a candidate donor population, subject to confirmation by demographic and genetic assessment.

### Morphometric variation

Morphometric analysis was possible only for certain localities, owing to the limited material collected, and revealed variability in shell size among populations. The largest shells were recorded in the Stalineshti River population (mean length *68.86 ± 2.52* mm; height *35.09 ± 1.12* mm; convexity *23.98 ± 0.89* mm); this population comprised individuals of several age classes.

The Murafa River population was of intermediate size (length *59.33 ± 2.08* mm; height *30.73 ± 0.63* mm; convexity *22.48 ± 0.88* mm; two 5-year-old individuals). The Sluch River population had the smallest shells (length *48.20 ± 2.24* mm; height *25.63 ± 1.09* mm; convexity *18.95 ± 0.87* mm) and the youngest composition (four of six specimens in the 4-year class).

The Styr River population was of intermediate size (length *53.00 ± 5.89* mm; height *27.63 ± 2.03* mm; convexity *22.17 ± 2.49* mm), with high internal variability, comprising mainly 6-year-old individuals (two specimens) and one 8-year-old.

The largest shells thus occurred in the Stalineshti River population. Larger shells tended to be associated with older individuals across the populations examined, consistent with continued shell growth with age; this relationship was not tested statistically because only population-level means were available for the four populations.

### Population grouping

**Fig. 6:**
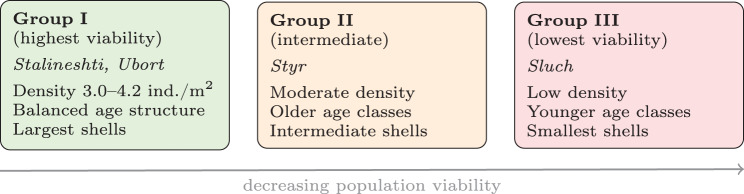
Grouping of *U. crassus* populations into three categories by comparative assessment of their combined demographic and morphometric indicators

By comparative assessment of their combined demographic and morphometric indicators (population density, age structure, maximum recorded age and shell dimensions), the populations were grouped into three categories (Fig. 6).

The first group (the Stalineshti and Ubort rivers) combined the highest densities (3.0–4.2 individuals/m$^2$), a relatively balanced age structure and the largest shells. The second group (the Styr River) showed intermediate density, a predominance of older age classes and intermediate shell size. The third group (the Sluch River) had the lowest density and the smallest shells, but included younger age classes.

### Temporal change in the Prypiat sub-basin

**Fig. 7:**
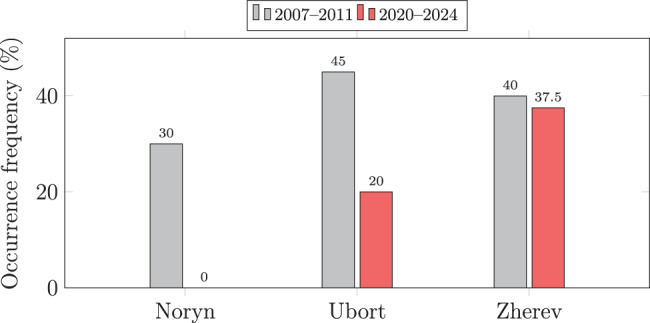
Changes in the occurrence frequency of *U. crassus* in river systems of the Prypiat sub-basin (2007–2011 vs 2020–2024)

Because the Prypiat sub-basin was identified as particularly important for the species, it was re-surveyed in 2020–2024 [17]. The overall occurrence of *U. crassus* in the sub-basin declined from 13 of 32 sites (40.6%) in 2007–2011 to 8 of 40 sites (20.0%) in 2020–2024; this difference was suggestive but did not reach statistical significance (Fisher’s exact test, odds ratio *=2.74*, *p=0.071*) (Fig. 7). At the river level, the species was not detected in 2020–2024 at the previously occupied Noryn River localities (a decrease from 30% to 0% of localities), whereas occurrence in the Ubort River fell from 45% to 20%. The Zherev River was comparatively stable (40% to 37.5%).

## Discussion

### Distribution and status in the right-bank Ukraine

The present, markedly mosaic distribution of *U. crassus* contrasts with its historically widespread occurrence across all river basins of Ukraine before the mid-twentieth century, and is consistent with the well-documented decline of the species across much of its European range [2, 9]. Recent genetic work has shown that the *U. crassus* complex comprises 12 European lineages, each with its own morphology and range [18]; the right-bank records studied here belong to *U. crassus* s. str. The highest frequency of occurrence in the Prypiat sub-basin supports the long-standing view, associated with the Ukrainian malacologist O. Korniushyn, that the Polissia region is of particular value for the conservation of unionid diversity in Ukraine [17], and identifies it as a priority area for the designation of Emerald Network sites [14].

### Co-occurrence and the indicator role of the species

The high co-occurrence of *U. crassus* with its congeners *U. pictorum* and *U. tumidus*, and its much weaker association with *A. cygnea* and the invasive *S. woodiana*, indicate broadly similar habitat requirements within the genus *Unio* and ecological separation from still-water taxa. At most occurrence sites (23 of 30; 77%) the species was found together with three or more native unionid species. Areas that concentrate the highest unionid species richness function as core areas of an ecological network – centres for the conservation of genetic, species and ecosystem diversity and potential donors for the restoration of biodiversity in adjacent territories. The habitats of *U. crassus* can therefore be regarded both as foci for the conservation of freshwater mollusc diversity and as practical indicators of well-preserved river reaches.

### Density in a European context

The per-site densities recorded in the right-bank Ukraine (basin means of 2–5 individuals/m$^2$, rarely up to 15) are low relative to the higher densities reported for some well-preserved Central and Southeastern European rivers, and to the much higher historical densities once recorded in Ukrainian rivers before the second half of the twentieth century. Such low densities are of conservation concern because they may fall below the threshold required for reliable fertilisation and recruitment in this gonochoristic, host-dependent species [7].

### Age structure, recruitment and host fish

The dominance of middle-aged individuals and the absence of individuals younger than two years across all sampled populations are consistent with limited recent recruitment, a pattern frequently reported for declining *U. crassus* populations elsewhere in Europe. Recruitment in this species depends critically on the availability of suitable host fish, into which the parasitic glochidial larvae must metamorphose; *U. crassus* actively releases larvae towards passing fish (“larval spurting”), which makes successful reproduction sensitive to the composition and abundance of the fish community [7]. We did not survey the fish assemblages of the studied rivers, so we cannot link the comparatively stable populations of the Ubort and Stalineshti rivers to host-fish availability; this is an important question for future work. We therefore recommend that monitoring of these candidate donor populations explicitly include assessment of host-fish availability and of juvenile recruitment.

### Anthropogenic pressures and temporal change

The apparent decline in occurrence in the Prypiat sub-basin, although not statistically significant with the present data, is consistent with the trajectory of *U. crassus* elsewhere and warrants concern, particularly the loss of detections in the Noryn River. Two broad classes of pressure are most likely involved: diffuse agricultural pollution (elevated nutrient and nitrate loads, fine-sediment input) and hydromorphological alteration (channel regulation and changes in the flow and thermal regime); both are known to influence the distribution of *U. crassus* [5]. Because we did not measure water chemistry, substrate or hydrology in situ, we cannot quantify the relative contribution of these drivers, and we treat them as hypotheses to be tested by targeted habitat monitoring. The comparatively resilient populations of the Prypiat-basin Uzh (Korosten–Narodychi) and Zherev rivers merit particular protection, while the Noryn and Ubort populations require active restoration measures.

### Conservation implications

Assessed against the criteria of the EU Habitats Directive (92/43/EEC) and the Water Framework Directive, the overall status of *U. crassus* in the right-bank Ukraine is of serious concern, despite the persistence of viable populations in some basins. Conservation effort should prioritise the protection of the residual populations of the Prypiat sub-basin and the maintenance of habitat connectivity. The populations of the Ubort (Sushchany) and Stalineshti (Mamalyha) rivers are the strongest candidate donor populations for future recovery programmes, subject to confirmation by demographic and genetic assessment; established European protocols for captive breeding and reintroduction [6, 11] and the recent roadmap for freshwater mussel conservation in Europe [9] provide a practical framework for such measures.

### Limitations

Several limitations should be borne in mind when interpreting these results. First, the numbers of occupied sites and of individuals available per population were small and uneven (down to single individuals in some basins), which limits the power of statistical comparison and the precision of demographic inference. Second, no in-situ environmental variables (water chemistry, substrate composition or hydrology) were measured, so habitat associations are inferred only qualitatively. Third, the fish assemblages were not surveyed, so the link between host-fish availability and recruitment could not be tested directly. Fourth, the 2007–2011 and 2020–2024 surveys differed in effort and in the set of localities examined, so the temporal comparison should be regarded as indicative rather than definitive. Finally, full morphometric analysis was possible only for a subset of populations. These constraints temper the strength of the conclusions, particularly regarding population decline, recruitment failure and the designation of donor populations, and define clear priorities for future, more intensive and standardised monitoring.

## Conclusions

Based on our analysis of *U. crassus* populations in the watercourses of the right-bank Ukraine, several conclusions relevant to the implementation of EU environmental directives can be drawn.

Our study identified the localities of highest conservation value for *U. crassus*. The population in the Ubort River (Sushchany) had the largest sample (19 individuals) and the most balanced age structure (4–7 years), consistent with comparatively favourable conditions, although the absence of individuals younger than two years indicates that recruitment may nonetheless be limited. The population in the Stalineshti River (Mamalyha) showed features of a stable adult population, including long-lived individuals (up to 8 years) and the largest shells. The younger Sluch River population (Baranivka) may also be relevant for species recovery.

The temporal comparison indicated a decline in occurrence in the Prypiat sub-basin between 2007 and 2011 and 2020–2024 (from 40.6% to 20.0% of sites; Fisher’s exact test, *p=0.071*), including the loss of detections in the Noryn River and a reduction in the Ubort River. Although this overall change was not statistically significant, the trend is consistent with continuing pressure on the species and highlights the need for conservation attention in these areas.

For effective *U. crassus* conservation by EU environmental directives, we recommend implementing a comprehensive conservation strategy including:

1. Establishing protected areas in the Ubort (Prypiat sub-basin) and Stalineshti (Danube basin) river basins as core conservation sites.
2. Developing long-term monitoring programs to track population dynamics and environmental parameters.
3. Investigating the factors affecting recruitment and juvenile survival, indicated by the absence of young individuals in all studied populations.
4. Creating river basin management plans that specifically address *U. crassus* conservation needs.
5. Designating the identified valuable populations as potential donor sources for future species recovery programs.

These data provide a scientific foundation for incorporating *U. crassus* conservation into Ukraine’s implementation of EU environmental directives, particularly the Water Framework Directive and Habitats Directive. They can inform the designation of new Emerald Network sites. The observed interspecific associations with other unionid species further underscore the importance of these habitats for overall freshwater biodiversity conservation.

Future research should focus on identifying the environmental factors supporting successful populations in the most promising localities, which will enable the development of effective conservation measures across the species’ range in Ukraine.

## Data Availability

The datasets generated during and/or analysed during the current study are available from the corresponding author on reasonable request.
